# Impact of service provision platforms on maternal and newborn health in conflict areas and their acceptability in Pakistan: a systematic review

**DOI:** 10.1186/s13031-015-0054-5

**Published:** 2015-08-22

**Authors:** Zohra S. Lassi, Wafa Aftab, Shabina Ariff, Rohail Kumar, Imtiaz Hussain, Nabiha B. Musavi, Zahid Memon, Sajid B. Soofi, Zulfiqar A. Bhutta

**Affiliations:** Division of Women and Child Health, The Aga Khan University, Karachi, Pakistan; Australian Research Centre for Health of Women and Babies, Robinson Research Institute, School of Paediatrics and Reproductive Health, The University of Adelaide, Adelaide, Australia; Head of Research and M&E, Greenstar Social Marketing, Karachi, Pakistan; Centre for Global Child Health, The Hospital for Sick Children, Toronto, Canada; Program for Global Paediatric Research, Hospital for Sick Children, Toronto, ON Canada

**Keywords:** Conflict area, Mothers and newborn, Maternal and neonatal health, Internally displaced population

## Abstract

Various models and strategies have been implemented over the years in different parts of the world to improve maternal and newborn health (MNH) in conflict affected areas. These strategies are based on specific needs and acceptability of local communities. This paper has undertaken a systematic review of global and local (Pakistan) information from conflict areas on platforms of health service provision in the last 10 years and information on acceptability from local stakeholders on effective models of service delivery; and drafted key recommendations for improving coverage of health services in conflict affected areas.

The literature search revealed ten studies that described MNH service delivery platforms. The results from the systematic review showed that with utilisation of community outreach services, the greatest impacts were observed in skilled birth attendance and antenatal consultation rates. Facility level services, on the other hand, showed that labour room services for an internally displaced population (IDP) improved antenatal care coverage, contraceptive prevalence rate and maternal mortality.

Consultative meetings and discussions conducted in Quetta and Peshawar (capitals of conflict affected provinces) with relevant stakeholders revealed that no systematic models of MNH service delivery, especially tailored for conflict areas, are available. During conflict, even previously available services and infrastructure suffered due to various barriers specific to times of conflict and unrest. A number of barriers that hinder MNH services were discussed. Suggestions for improving MNH services in conflict areas were also laid down by participants.

The review identified some important steps that can be undertaken to mitigate the effects of conflict on MNH services, which include: improve provision and access to infrastructure and equipment; development and training of healthcare providers; and advocacy at different levels for free access to healthcare services and for the introduction of the programme model in existing healthcare system. The obligation is enormous, however, for a sustainable programme, it is important to work closely with both the IDP and host community, and collaborating with the government and non-government organisations.

## Background

Forced internal displacement of people is a tragedy for those who experience it. Whether it ensues as a result of natural disasters, political or armed conflict, or human rights violations, the displacement results in a number of devastating outcomes to one’s identity, family and livelihood. It is estimated that 28.8 million people were internally displaced as a result of international and internal armed or political conflicts at the end of 2012 [1]. Sub-Saharan Africa alone hosts nearly 10.4 million of the internally displaced persons (IDPs), with most displacements attributed to conflicts in Syria and Democratic Republic of Congo (DRC). In South and South-East Asia the number of IDPs grew from 2.7 million in 2005 to 4.6 million in 2010, attributable to a rise in the number of IDPs in Pakistan, Afghanistan, Philippines, Myanmar, India and Indonesia, where most people were displaced by on-going armed conflicts [1].

In Pakistan, the number of IDPs were 3 million in 2009 [1]. This was due to the significant population movement experienced as a result of government operations against non-state armed groups as well as sectarian violence in Khyber Pakhtunkhwa (KP) and Federally Administered Tribal Areas (FATA) in 2008. Estimates from 2012 show that the numbers have stabilised in Pakistan with the return of 3.6 million IDPs to their homes in KP and FATA, yet 412,000 new IDPs were registered in the year 2012 [1]. Despite the on-going armed conflict and the health related issues of IDPs in Pakistan, the efforts to stabilise conditions continue. UNICEF has reported that in December 2012, 83,867 children were vaccinated against polio during a supplemental National Immunisation Campaign. In 2012, 127,311 children and 55,566 pregnant and lactating women screened for acute malnutrition as well [2]. Approximately 9,435 children (50 % girls) have also been enrolled in 44 UNICEF education centres [2].

**Fig. 1:**
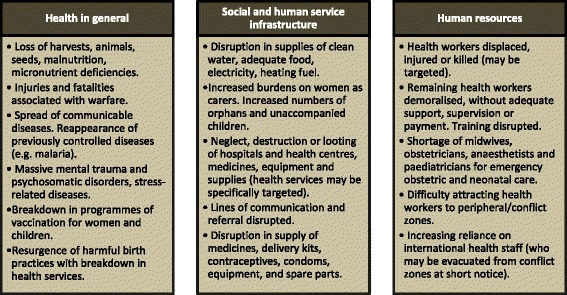
Impact of armed conflict on maternal and newborn health related issues. Source: WHO 2000^3^

**Fig. 2:**
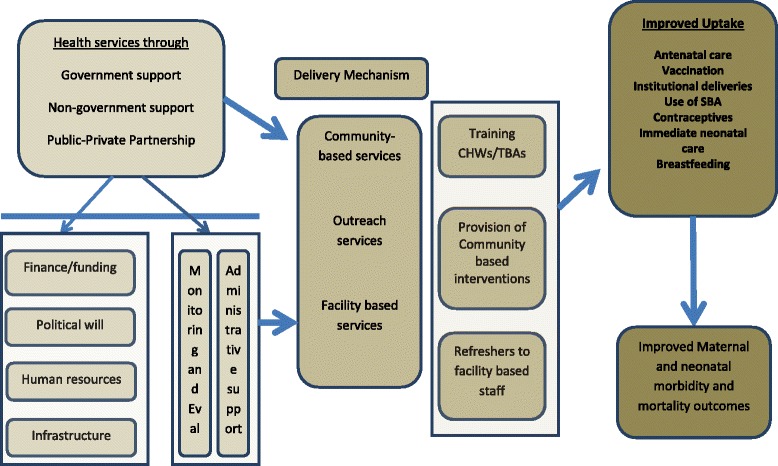
Illustrative model of service delivery in conflict areas

According to WHO [3], armed conflicts can result in a number of effects on health, health service infrastructure and human resources (Fig. 1). The conditions in conflict areas are ideal for disease and trauma to proliferate and in such scenarios, women and children in particular are at high risk. Moreover, it is difficult to deliver effective health services, as organisations, institutions, and resources are adversely affected by conflict and the political instability that surrounds and follows it. The unavailability of healthcare providers (especially female), unavailability of drugs and resources, disease outbreaks and even challenges in maintaining basic hygiene have a high impact on MNH (maternal and newborn health) outcomes. Although international and local non-governmental organisations (NGOs) continue to work and deliver health services in security compromised areas in Pakistan [4], there is a need to develop further understanding of how to address the MNH needs in Pakistan’s conflict areas. Various models and strategies have been implemented over the years in different parts of the world to improve maternal and newborn health in conflict affected areas [1–3]. These strategies are based on specific needs of the communities and whether they can be accepted by the locals. In order to evaluate the delivery mechanisms we have derived an illustrative model for service delivery in conflict areas (Fig. 2). The framework suggests delivery mechanisms which allow improving delivery of healthcare services by training of community health workers who in turn can provide key healthcare services to the community, including vaccination, medications, awareness and guidance and identification of areas of need.

The specific objectives of this paper were to undertake a systematic review of global and local (Pakistan) information from conflict areas on platforms of health service provision implemented at community and/or facility level to improve MNH within the last ten years (September 2003 to September 2013). Furthermore, to interview the stakeholders from Pakistan to understand the acceptability of effective models of service delivery in conflict areas. Lastly, based on the information from the systematic review and information from stakeholders, the paper proposed key recommendations for improving the coverage of health facilities in those areas.

## Methods

### Data sources and searches

The studies were searched on The Cochrane Library, Medline, PubMed, Popline, CINAHL, Google and relevant international bodies’ websites, NGOs and Inter-Governmental Organisations (IGOs) (UNFPA, UNICEF, SAVE the Children) and ministries of health. Detailed examination of cross-references and bibliographies of available data and publications to identify additional sources of information was also performed. The following basic illustrative search strategy was adapted for the various databases and search engines: [(“maternal and newborn health*” OR “health service*” OR “service delivery” OR “health models*” OR “health worker*” OR “community health worker*” OR “lady health worker*”) AND (“conflict area*” OR “internally displaced people*” OR “IDP*” OR “displaced population” OR “fragile area*)]. Language restrictions were not applied and MeSH terms were used. The outcomes of interest included maternal and neonatal health, morbidity and mortality. The last date of search was 10^th^ September 2013. The review only focused on service delivery methods devised and implemented in the last decade (September 2003 to September 2013). These dates were selected by the advisory committee of experts established by ministry of health, Government of Pakistan to review the latest services delivery mechanism in Pakistan and globally so that recommendations could easily be articulated into the existing health situation.

### Study selection

All available evidence for the impact of platforms of health service delivery in conflict areas was systematically analysed. The review included studies where services were provided to conflict affected population and these services were evaluated with some form of control group or with baseline indicators. In order to understand these interventions and programmes better the review also included case studies, programme evaluations and descriptive cross-sectional analyses where a comparison arm was not used. However, studies related to refugees were excluded because there are many studies on refugee immigrants from high income countries where they are provided with health services similar to local residents and therefore, we cannot draw information which could relate to health care delivery mechanism in conflict affected areas in developing countries.

### Screening process and expert opinions

Titles and abstracts were screened by two abstractors and any disagreements on selection of studies were resolved by the third reviewer. On meeting the eligibility, each study was double data abstracted into a standardised form. Interviews with various stakeholders (public, private, and international organisations dealing with MNH issues in conflict areas) were also conducted in Balochistan, FATA and KP provinces of Pakistan in two consultative workshops. The discussions explored the current level of MNH services in conflict areas; various barriers to MNH services in conflict areas; and the potential of various models for improving access to MNH services. The consultative meetings were recorded and transcribed. The data was analysed in terms of categories, themes, and patterns.

Taking into consideration the systematic review findings, key stakeholders were consulted at provincial and district levels in Pakistan to gather key information on acceptability/adoptability of effective service delivery models in conflict areas and future policy implications. Finally based on the results of literature review and consultative meetings, recommendations were made for MNH service delivery in Pakistan’s conflict areas to achieve increased coverage and accelerate progress towards attainment of MDGs 4 and 5.

## Results

### Literature review

**Fig. 3:**
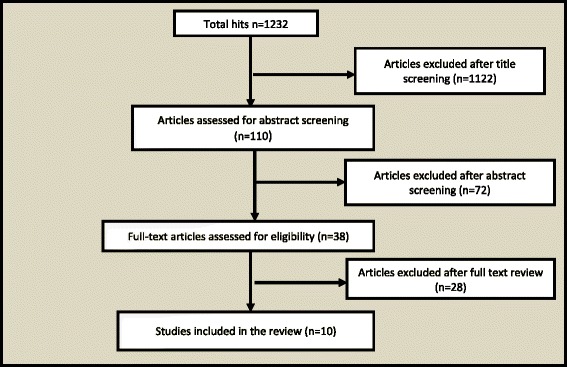
Illustrative model of service delivery in conflict areas

The literature search revealed 10 studies [2, 5–14] that evaluated the impact of strategies for MNH service delivery in conflict areas (Fig. 3). These studies were from Afghanistan [5, 6], Pakistan [2, 14], Myanmar (Burma) [11], Sudan [9], Tanzania [12], Liberia [8], Guatemala [10], and the DRC [7, 13]. The review did not come across any randomised control trials or studies comparing intervention with a control arm. A few of the included studies were narratives with no quantitative data, but were included to highlight different strategies of healthcare used in effort to improve MNH. As a result, meta-analysis could not be performed. A historical summary of the conflicts surrounding the countries included in this analysis are described in Table 1. The included studies are described in detail in Tables 2 and 3. The platforms for health delivery mechanisms in the included studies have been categorised into community based services, outreach services and facility based services as described in the framework (Fig. 2).

**Table 1:** Historical events that led to conflicts in some of the countries included in our analysis

Afghanistan [6]
The era of armed conflicts started in Afghanistan with the Soviet army invasion in December 1979 to support the Communist government. Although the Soviets left in 1989, Afghanistan has remained in the grip of violence as a result of various political and religious conflicts. In 2001, a new international conflict developed as a result of war against terrorism. In the 1990s the Mujahidin and the Taliban forces were constantly at war in their struggle for power. After the Taliban took control, the war against terrorism was aimed at driving the Taliban forces out of Afghanistan. This led to another prolonged era of war and conflicts leading to suffering for Afghan people.
The war resulted in considerable destruction of infrastructure. In rural areas, whole villages were destroyed together with their orchards, irrigation systems and fields. A million people are said to have died and 700,000 women were widowed by the end of the war. By 1989, there were 3.7 million documented refugees in Pakistan and almost three million in Iran. Huge numbers of people were internally displaced within the country. A large proportion of professionals, including health professionals, and many other educated people left the country.
The Democratic Republic of Congo [17]
During the Congo wars from 1996 to 1997 and 1998 to 2003, the conflict involved nine countries and more than 40 rebel groups. Today three main categories of armed groups operate in eastern Congo: the Rwandan Hutu FDLR; the Rwanda and Uganda-backed M23; and various local armed “Mai Mai” groups. In addition, the Congolese army has committed many human rights abuses. All of these groups have attempted to seize control of natural resources in order to continue fighting. The conflicts begin after the outpouring of refugees into DRC as a consequence of the ‘Rwanda genocide’. These refugees formed a rebellious group and led to the first and second Congo wars when the government of DRC decided to purge all Rwanda elements from the system.
Since the beginning of 2012, ethnic tensions and inequitable access to land have led to renewed violence in the east and north-east of DRC resulting in the displacement of more than 2.2 million people inside the country. In addition, almost 70,000 people have crossed the border into neighbouring Rwanda and Uganda.
At the same time, in the first half of 2012, some 15,000 refugees from the DRC returned home, mainly to Equateur province. Their reintegration will be supported by UNHCR through community-based projects and targeted assistance to individuals to enhance their livelihoods. More than 400,000 Congolese refugees currently remain outside the DRC. Since the beginning of the conflicts over 5.4 million people have died and over two million have been displaced.
Sudan [18]
Since gaining independence from Britain and Egypt in 1956, Sudan has experienced more years of conflict than peace. The first civil war, from 1955 to 1972, was between the Sudanese government and southern rebels who demanded greater autonomy for southern Sudan. The war ended with the 1972 Addis Ababa Agreement, which granted significant regional autonomy to southern Sudan on internal issues. The second civil war erupted in 1983 due to longstanding issues heightened by then President Jaafar Nimeiri’s decision to introduce Sharia law. Negotiations between the government and the Sudan People’s Liberation Movement/Army (SPLM/A) of southern Sudan took place in 1988 and 1989, but were abandoned when General Omar al-Bashir took power in the 1989 military coup. Bashir remains president of Sudan today. These internal ensions drove the country’s decades-long civil war, which led to South Sudan’s secession from Sudan on July 9, 2011. Despite this turn of events, numerous internal conflicts continue in Sudan and South Sudan.
The war has left two and a half million people dead and four million people displaced.
Pakistan [19]
Pakistan comprises five broadly distinct regions: Punjab in the north-east, Gilgit-Baltistan and Azad Kashmir in the north, Sindh in the southeast, Balochistan in the south-west, and Khyber Pakhtunkhwa (KP) province and the Federally Administered Tribal Areas (FATA) which border Afghanistan in the Pashtun north-west. Since its creation in 1947, Pakistan has experienced alternating periods of civilian and military rule. Pakistan faces enormous challenges on a range of fronts, including security and terrorism, sectarian and ethnic violence, a troubled economy and recurrent natural disasters. Military intervention by the US and NATO in neighbouring Afghanistan since 2001 and Pakistan’s alignment with the US against al-Qaida and the Taliban has fomented opposition to the government. Islamist armed groups seek to overthrow tribal governance structures in the north-west and the government has struggled to maintain law and order. Indiscriminate suicide attacks, the use of improvised explosive devices, targeted killings and intimidation by non-state armed groups continue, claiming more than 360 civilians’ lives in KP alone in 2012. Military operations against non-state armed groups, most notably Tehrik-eTaliban Pakistan, have escalated since 2007.
An estimated five million people have been displaced by conflict, sectarian violence and wide-spread human rights abuses in the north-west as a whole since 2004. Today, Pakistan faces a renewed displacement crisis fuelled by massive new forced population movements in FATA, the current focus of conflict in the region. More than 415,000 people were newly displaced in 2012 alone.
Guatemala [20]
Guatemala is a mainly mountainous country in Central America. When Spanish explorers conquered this region in the 16th century, the Mayans became slaves in their own homeland. They are still the underprivileged majority of Guatemala’s population. Civil war existed in Guatemala since the early 1960s due to inequalities existing in economic and political life. In the 1970s, the Maya began participating in protests against the repressive government, demanding greater equality and inclusion of the Mayan language and culture; ultimately resulting in a guerrilla movement. In 1980, the Guatemalan army instituted “Operation Sophia,” which aimed at ending insurgent guerrilla warfare by destroying the civilian base in which they hid. This programme specifically targeted the Mayan population, who were believed to be supporting the guerrilla movement.
Over the next three years, the army destroyed 626 villages, killed or “disappeared” more than 200,000 people and displaced an additional 1.5 million, while more than 150,000 were driven to seek refuge in Mexico. The violence faced by the Mayan people peaked between 1978 and 1986.
After 36 years, the Guatemalan armed conflict ended in 1996 when the government signed a peace accord (the Oslo Accords) with the insurgent group, the Guatemalan National Revolutionary Unity.
Myanmar (Burma) [21]
Myanmar (aka Burma) has been in a state of constant civil war since independence in 1948. Myanmar is one of the most ethnically-diverse countries in the world with key non-Burma ethnic groups demanding equality with the Burmans in the three public realms, specifically the protection of ethnic culture, language, and religion, the devolution of tangible executive, legislative, and judicial power to the ethnic states within a true federal union, and a democratic form of government. With their demands unmet, the ethnic groups turned to armed insurgency. The civil war and the perceived threat of secession by ethnic states from Myanmar led in 1962 to a military coup. Since then, the military has dominated the affairs of the country seeing itself as the sole force capable of holding the country together.
The major non-Burman ethnic groups are the Arakanese, Chin, Kachin, Shan, Karenni, Karen, and Mon, all of which have their own states in which they are the dominant ethnic group. All these states have ethnic insurgent activities of varying intensities against the Myanmar military (aka Tatmadaw). The Tatmadaw has been employing a counterinsurgency strategy which attempts to deny the ethnic insurgents access to food, funding, information, and recruits. Also the Myanmar regime policies have led to the impoverishment of and human rights abuses toward the ethnic peoples leading hundreds of thousands of them to seek safety in adjacent countries – Thailand, China, India, and Bangladesh, through resettlement in other countries, and as internally displaced persons in the jungle inside Myanmar.
Currently there are at least 450,000 IDPs living all over Myanmar. There were 166,000 new IDPs registered alone in 2012.

**Table 2:** Characteristics of included studies

Author	Country	Study design	Delivery mechanism	Description of methods/intervention	Results	Quality assessment	Notes
UNICEF [2]	Pakistan	Case Study	Community based services: In coordination with department of health and WHO.	UNICEF supports maternal and child healthcare services including provision of around-the-clock basic emergency obstetric services through skilled birth attendants. Community outreach workers/social mobilisers and health educators have conducted awareness sessions on infant and young child feeding, community-based management of acute malnutrition and hygiene education.	6,458 children were reached with lifesaving immunisations during a national immunisation campaign.	N.A	This is a case study of situation of armed conflict affected areas in Pakistan.
					72,193 persons are benefitting from UNICEF supported provision of 255,000 l of safe drinking water per day		
Aitken [6]	Afghanistan	Case Study	Community based services: Training of CHWs as part of the Basic Package of Health Services. This was sponsored by NGOs, UNAID and World Bank.	The Basic Package of Health Services consists of various aspects including facility and community based approaches. In this case study only CHW training has been discussed. Specifics on programme implementation or methodology used to collect data have not been mentioned in the report. The differences in skilled birth attendance and antenatal care three years after the introduction of CHWs in community are given.	Skilled birth attendance rose from 7 to 19 %. Antenatal care use increased from 8 to 32 %	N.A	This is a case study of situation of Afghanistan between 2003 and 2006 and how health parameters have changed with time. The paper describes the efforts of different organisations and their funding. Little information is available about the interventions used.
Miranda [10]	Guatemala	Pre-Post Surveys	Outreach service: Safe motherhood was advocated through a mobile healthcare unit in 23 rural frontier communities. The mobile team was responsible for training of community health workers, community education and the provision of maternal health services. The programme was carried out by Marie Stopes Mexico	Evaluation of the services provided by mobile unit was conducted using pre and post KAP surveys in 12 selected communities using a representative sample. Interviews were conducted with 388 indigenous men and women of reproductive age in the baseline survey in June 2001 and with 398 in the post-intervention survey in June 2003. Further details of the methodology are not available.	Prenatal and childbirth care by midwives increased significantly from 71 to 89 % (*P* = 0.00)	Please refer to Table three	This paper was presented in the RHRC conference proceeding in 2003. Only the abstract is available and details ofmethodology and results are not clear.
							Marie Stopes Mexico is an NGO focusing care towards reproductive and maternal health.
Mullany [11]	Myanmar	Pre-post Surveys	Community based services: CHWs, TBAs and maternal health workers were trained for eight months and allowed to work in the community for two years.	Two-stage cluster-sampling surveys among married women of reproductive age (15–45 y) conducted before and after programme implementation enabled evaluation of changes in coverage of essential antenatal care interventions, attendance at birth by those trained to manage complications, postnatal care, and family planning services.	Skilled birth attendance increased from 5.1 to 48.7 %	Please refer to Table three	N.S
Wabulakombe [13]	Democratic Republic of the Congo	Unclear	Outreach services: A safe motherhood and family planning programme to reduce maternal and infant mortality was conducted after a survey to identify the healthcare needs of the community. Sponsored by Merlin charity.	Details of the methodology and analysis are not provided.	Antenatal consultation rate increased from 55 to 88 %. The proportion of safe deliveries conducted by the trained staff increased from 37 to 60 %. Maternal mortality decreased from 0.22 to 0.15 %	N.A	This paper was presented in the RHRC conference proceeding in 2003. Only the abstract is available and details on the methodology and results are not clear.
				Programme activities included: Raising community awareness, making the health facilities operational, transferring skills to the district health team, changing the health-related behaviour of the population, providing drugs and equipment to health facilities			
Casey [7]	Democratic Republic of the Congo	Case study	Facility based services: Evaluation of nine EmONC centres	Nine EmONC centres evaluated as providing inadequate healthcare were brought to attention of Ministry of Health. These centres were then supplied with resources and equipment.	No analysis was performed	N.A	N.S
UNICEF [5]	Afghanistan	Case Study	Community based services for health, nutrition and hygiene. UNICEF	Micronutrient supplementation, exclusive breastfeeding and complementary feeding. Children, women and communities displaced by emergencies will have improved access to maternal, infant and child health services at the community and facility levels. Measles vaccinations will be provided to all children up to 15 years of age and children under 5 will receive vitamin A supplementation.	The construction of 1,200 community water systems, including 1,100 borehole hand pumps and 100 small pipe water systems will provide access to safe drinking water for more than 30,000 families.	N.A	This project aimed to improve MNCH in Afghanistan
Rutta [12]	Tanzania	Descriptive cross sectional	Facility based services: Through a healthcare centre, community sensitisation to HIV, trainings of healthcare workers, voluntary counselling and HIV testing, infant feeding, counselling, and administration of Nevirapine were advocated.	Two year data from four antenatal clinics and two hospitals’ delivery registers was used for descriptive analysis.	92.3 % of the pregnant women who received counselling at these centres agreed to go through HIV screening. 93 % of the women tested positive for HIV agreed on Nevirapine. All of the infants of HIV positive mothers delivered were given Nevirapine soon after birth.	N.A	N.S
				Main outcome measures include: HIV testing acceptance rates, percentage of women receiving post-test counselling, Nevirapine uptake, and HIV prevalence among pregnant women and their infants.			
McNab [9] (publication year not clear)	Sudan	Case study	Facility based services: the setting up of an EmONC centre which allowed free of cost RH and maternal health services. This was sponsored by the Ministry of Health Sudan and the ARC.	The report describes how the EmONC centre was set up and how it could be of benefit to the community. No analysis or data collection was performed.	No analysis was performed	N.A	N.S
McGinn [8]	Liberia	Case study	Facility based services: American Refugee Committee procured and supplied obstetric equipment and drugs, recruited and trained national staff and upgraded three hospitals to provide comprehensive obstetric care, and six health centres to provide basic emergency obstetric care. In addition, ARC employed a Nigerian surgeon to work at the remote Grand Gedeh County Hospital, and trained surgical technicians.	Very short narrative of the implementation of this programme is given.	No analysis was performed	N.A	This programme was shut down due to increased conflicts in 2004 before the impact could be evaluated.
				ARC set out to strengthen family planning and improve emergency obstetric care in Montserrado, Grand Gedeh and Sinoe counties.			

*ARC* american refugee committee, *CHWs* community health workers, *EmONC* emergency obstetric and neonatal care, *HIV* human immunodeficiency virus, *N.A* not applicable, *NGOs* non-government organisation, *N.S* not significant, *RHRC* reproductive health response in conflict, *TBAs* traditional birth attendants

**Table 3:** Quality assessment of studies included with a pre-post study design based on criteria by Loevinsohn [15]

Quality assessment criteria for pre-post studies without control arm			
Study features^a^	Assessment	Miranda [10]	Mullany [11]
Study based on explicit theory	Yes/No/Unclear	Yes	Yes
Adequate description of how educational strategy adapted to local conditions	Yes/No/Unclear	Unclear	Yes
Example given of materials or educational process	Yes/No/Unclear	Unclear	Yes
Adequate description of resources required to carry out interventions	Yes/No/Unclear	Unclear	Yes
Measure outcome before and after intervention	Yes/No/Unclear	Unclear	Yes
Measurement method same before and after	Yes/No/Unclear	Unclear	Unclear
Period between education and outcome more than one year	Yes/No/Unclear	Yes	No
Author claimed positive results for interventions	Yes/No/Unclear	Yes	Unclear
Paper included discussion of possible biases and caveats (or limitations)	Yes/No/Unclear	Unclear	Unclear
Paper included *P* values or confidence interval	Yes/No/Unclear	Yes	No
Analysis employed some form of modelling such as regression	Yes/No/Unclear	Unclear	Unclear
Exposure to intervention monitored	Yes/No/Unclear	Yes	Yes

^a^Adopted from Leovinsohn 1990

### Community based services

Community-based services were used as a delivery mechanism in two studies. These studies provided data from programmes and interventions carried out in Afghanistan [6] and Eastern Burma [11]. Training of CHWs, conducting awareness workshops and empowering (using strategies such as information, education and communication) community members to tackle issues of MNH were widely used methods. Results showed that utilisation of these services had the greatest impact on skilled birth attendance (SBA) and antenatal consultation rates. Results from different studies evaluating such programmes in Eastern Burma [11] and Afghanistan [6] showed that SBA increased between 12 and 42 % [6, 11] and antenatal consultation rates increased by approximately 33 % [11].

### Outreach services

Outreach services have been used as a delivery mechanism in one of the programme evaluations conducted in DRC [13]. The study evaluated people’s knowledge, attitudes and behaviours regarding safe motherhood and family planning after implementation of a safe motherhood programme. The programme involved an outreach service, consisting of trained physicians and CHWs, with activities including raising community awareness, making the health facilities operational, transfer skills to the district health team, changing the health-related behaviour of the population and providing drugs and equipment, overcoming religious and cultural obstacles, and providing motivation to health facilities by focus group discussions and home visits. The study showed that antenatal consultation rate increased from 55 to 88 % while the proportion of safe deliveries conducted by the trained staff increased from 37 to 60 % since the beginning of the reproductive health programme. Maternal mortality also decreased from 0.22 to 0.15 %. A pre-post survey evaluating outreach MNH and reproductive health services to an IDP camp in Guatemala [10] showed that prenatal and childbirth care by midwives increased significantly from 71 to 89 %. The mobile unit also trained CHWs in the community and increased awareness regarding healthcare services.

### Facility based services

The strategies employed at facility level were the basis of a few studies. These strategies include upgrading infrastructure, provision of drugs and supplies, etc. These studies were from Sudan [9], Tanzania [12], Liberia [8], and Democratic Republic of the Congo (DRC) [7]. The study from Sudan highlighted the setting up of an emergency obstetric and newborn care (EmONC) centre and the issues and hurdles relating to its functioning [9]. The study from DRC highlighted inadequate healthcare provided by EmONC centres there [7]. These centres were then supplied with resources and equipment and results showed the importance of improving the infrastructure of facility based services as delivery mechanisms. A report from Liberia described how American Refugee Committee (ARC) in 2001 procured and supplied obstetric equipment and drugs, recruited and trained national staff and upgraded three hospitals to provide comprehensive obstetric care, and six health centres to provide basic emergency obstetric care [8]. In addition, ARC employed a Nigerian surgeon to work at the remote Grand Gedeh County Hospital, and trained surgical technicians. The surgeon performed an average of three Caesarean sections per month. Local project costs were less than $1,000 per month. This programme was shut down due to increased conflicts in 2004 before the impact could be evaluated.

The EmONC centres setup in Tanzania were evaluated and showed significant impact on MNH. The study described the results of a two-year pilot programme implementing prevention of mother to child HIV transmission by counselling, training health workers, encouraging HIV testing and use of Nevirapine [12]. 92.3 % of the pregnant women who received counselling at these centres agreed to go through HIV screening. Furthermore, 93 % of the women who tested positive for HIV agreed on Nevirapine. All of the infants of HIV positive mothers delivered in the two hospitals were given Nevirapine soon after birth.

### Combination of community-based and outreach services

UNICEF has been providing healthcare services in many conflict hit areas around the world. The delivery mechanisms for healthcare services are various. They provide education and training for the community to manage issues relating to hygiene, sanitation and nutrition. They also built up facilities and centres for managing issues related to nutrition and MNH. An example is the efforts of UNICEF for three IDP camps in FATA areas of Pakistan [2]. In 2012, 127,311 children and 55,566 pregnant and lactating women were screened for acute malnutrition. Furthermore 1 million people (including returnees) have benefitted from UNICEF-supported water, sanitation and hygiene (WASH) items including hygiene kits, plastic buckets, and jerry cans at all three camps. 90,094 people (55 % female) at the three camps had access to 6,011 latrines, 2,889 washrooms, 1,551 washing pads and 407 solid waste collection points [2]. This improves the overall health status of the women living at the camps and thus indirectly improves pre-pregnancy and pregnancy related outcomes. Similarly UNICEF is working in Afghanistan to provide 108,000 pregnant women with safe delivery and newborn care kits [5].

### Consultative meetings

Discussions revealed that no systematic models of MNH service delivery, tailored for conflict areas, are functional in Pakistan. The following themes emerged from the discussions.

### Barriers to MNH services provision in Pakistan’s conflict areas

A fundamental concern among stakeholders was that MNH and other health services are not high enough on the government’s agenda in conflict affected areas. An environment of uncertainty and insecurity is considered to be the biggest hurdle to MNH services accessibility. Lack of appropriate and safe transportation and disruption of communication networks compound these problems.

Severe dearth of skilled health professionals in conflict areas is the greatest health system specific barrier. Skilled health staff commonly moves away from conflict areas due to insecurity and deterioration in basic amenities. Although local community health workers usually stay in the area, even their mobility is restricted. Limited availability of commodities, supplies, equipment and frequent power shortages also hinder service provision.

Lastly, in most conflict areas in Pakistan, conservative cultural norms do not allow women to travel unaccompanied. Presence of male providers discourages women from utilising services while trained female providers are usually not available. Further, in ethnically Pashtun dominated areas, NGOs are considered un-Islamic, limiting their role.

### Applicability of various service delivery mechanisms in conflict situations

Community based services are considered favourable in terms of good access, 24/7 availability, cost effectiveness and acceptability, especially if local providers are available. A strong community based component is considered imperative in any MNH services model in conflict areas. Facility based services offer advantages of skilled providers in a comfortable environment, with required commodities and equipment. However, access could be a major issue in periods of conflict. Non-availability of 24/7 services at BHUs and RHCs would also be a barrier. Mobile services offer a good combination of ease of access and quality services. These services can be offered in areas with high level conflict, where infrastructure has been damaged or distances are too great to travel to facilities. Their cost is the major barrier to large scale use. Prioritisation for the most highly affected areas could be a viable strategy.

### Role of non-state actors in disrupting health services

Non-state actors have the ability to profoundly disrupt travel, security and health services in conflict areas. Participants felt that engaging them to allow safe passage to health workers on humanitarian basis is not being adequately explored.

### Suggested features of an MNH service delivery model in Pakistan’s conflict areas

Antenatal, natal and postnatal services, including EmOC; nurseries for newborns; immunisation and nutrition services were considered vital. Psychological support services were also suggested.

### Facilitation during repatriation

It was stressed that ensuring quality MNH services in post-conflict areas is critical for smooth repatriation of IDPs.

### Recommendations

A number of possible interventions emerge from the literature review and group discussions. A conducive political environment is essential for improving health services in conflict areas. Provincial strategies for MNH in conflict affected areas, developed in consultation with government and non-government stakeholders, would lay down a practical basis. Decisions regarding stewardship of the processes of providing MNCH care to displaced populations, whether by the government or other non-governmental, international or intergovernmental agencies, should be made early as conflict and displacement start. In any case, early technical guidance should be sought from international and intergovernmental organisations that have specific experience in providing MNH health services to conflict affected populations considering the often limited expertise available within the government and administration. To avoid loopholes in service delivery, the roles of various government agencies and geographical areas of responsibility in times of conflict must be clearly delineated, particularly regarding continuity of services to IDPs moving from one province/territory to another. Another political imperative is to ensure separation of health services from the political dimensions of the conflict, especially when the state is an active party. Negotiations should be held with non-state actors to allow safe passage of health workers, women, children and supplies on humanitarian grounds. The role of various international and UN agencies could be crucial in providing essential care in these circumstances if their presence is considered impartial and non-political by non-state actors [15].

Retaining and facilitating skilled health workers in conflict areas would require a multi-pronged approach. Institution of special incentive packages, enhanced security and task shifting to local health workers accompanied by appropriate training could be effective measures. Even though current literature is ambivalent about the role traditional birth attendants should play during conflict situations, in the absence of other providers they can be utilised given their availability and trust in communities [7, 11, 15], especially in the context of poorly developed areas in developing countries. The role of community level health providers should focus on early recognition of complications in pregnancy, delivery and neonate. They can also help families in preparation and planning for birth, education about danger signs in mother and child, in providing clean kits for delivery at home and perform normal deliveries [11, 15]. However, it must be remembered that TBAs cannot be relied upon to provide care in case of complications [8]. Therefore, community providers’ services should be complemented by availability of facilities able to provide emergency obstetric and neonatal care. To strengthen local health services, temporarily decentralisation would allow local health institutions to tailor services to local conditions [16]. Collaboration with international agencies could also be helpful in filling some health HR gaps. Given conservative cultural norms in most conflict affected areas in Pakistan, induction of female health workers is a powerful way of ensuring women and children’s access to these services as has been witnessed in Afghanistan [6].

In the instances where records are available, MNH services provided to displaced populations have often not been comprehensive enough. To be effective in containing or reducing mortality and morbidity, services need to be broad enough including prenatal, delivery and postnatal services including facility based care for complications [11]. Further, reliable availability of contraceptives is one of the most effective ways of reducing maternal and child mortality from planned and unplanned pregnancies. Finally, availability of necessary medical supplies and equipment is needed for these interventions to be effective [7].

Successfully financing these services would require additional regular budgetary support, especially for areas with longer term conflict. Budgetary gaps could be filled with the help of international organisations or philanthropist groups. Adequate consideration must also be accorded to monitoring of MNH situation by either third party or combined monitoring by the government and other organisations working in the area based on relevant District Health Information System indicators. Finally, as the conflict abates, rapid evaluation and reconstruction of health infrastructure must precede or accompany repatriation of IDPs.

### Research gaps

High quality experimental designs are required that report outcomes measured at similar scales to be pooled together. Secondly, there is a dearth of information on contextual characteristics of implementation strategies. Reporting of such information would play an important role in replicating successful activities in other conflict areas. Operational research on how to deliver these services in conflict affected areas equitably and with cultural competence is needed.

Most research we found has been done among IDPs residing in stable camp settings. However, significant numbers of IDPs choose not to live in camps and parts of population stay on in areas of on-going conflict. There are significant differences in health status and possible modes of service delivery between displaced persons living in camp and non-camp environments; with conditions often more precarious for non-camp populations [8]. Effective ways of delivering MNCH interventions to IDPs in non-camp settings and to people who choose not to leave conflict-affected areas is a crucial, although challenging, area that needs further research.

## Conclusion

During conflicts and emergency situations, conflict affected populations are susceptible to poor health and infectious disease outbreaks. Women and children are particularly vulnerable to these health effects. In Pakistan’s conflict affected areas even those services which were previously being provided tend to get attenuated due to damage to infrastructure, insecurity, transportation barriers and migration of health staff. Our review suggests that in conflict areas, the condition of local health infrastructure, the terrain, and acceptability in the local population would determine the appropriate service delivery mechanisms. The type of mechanisms used would have to be tailored according to prevailing conditions and reviewed as circumstances change. Government and other stakeholders need to synchronise their efforts to ensure provision of HR, funding, infrastructure, equipment and supplies for MNH services in these areas. Achieving that would help in limiting maternal and neonatal morbidity and mortality in the country’s conflict affected areas.
